# Traumatic Page Kidney Induced Hypertension in Critical Care: Immediately Resolved or Long-Term Resistant Problem

**DOI:** 10.1155/2013/201424

**Published:** 2013-07-17

**Authors:** E. Brotfain, L. Koyfman, A. Frenkel, A. Smolikov, A. Zlotnik, M. Klein

**Affiliations:** ^1^Department of Anesthesiology and Critical Care, Soroka Medical Center, Ben-Gurion University of the Negev, 85102 Beer Sheva, Israel; ^2^Department of Radiology, Soroka Medical Center, Ben-Gurion University of the Negev, 85102 Beer Sheva, Israel

## Abstract

Page kidney is a well-known phenomenon causing hypertension, due to compression of renal parenchyma by a subcapsular hematoma, of either traumatic or non-traumatic origin. The main therapeutic approach is based on surgical approach (nephrectomy or hematoma evacuation) and antihypertensive treatment. In this paper we present a post-traumatic case of Page Kidney in a Critical Care unit. We discuss different therapeutical opportunities to extremely elevated systemic blood pressure resistant to traditional drug therapy.

## 1. Introduction

Page kidney is a well-known hypertensive phenomenon caused by compression of renal parenchyma by a subcapsular hematoma [1, 2]. Both traumatic [3–5] and spontaneous bleeding etiologies have been reported [1]. The main mechanism responsible for development of resistant hypertension is believed to be renin-mediated [1]. Renal mass lesions and invasive procedures complications may also result with clinical features of Page kidney. 

The initial management may be divided between antihypertensive treatment only and a combine surgical approach based on drainage of the subcapsular renal hematoma and in extereme cases total nephrectomy [1, 2].

In this paper we describe the case of acute posttraumatic Page Kidney syndrome in a multiple trauma critically ill patient.

## 2. Case Report

An 18-year-old, previously healthy male was admitted to our intensive care department after suffering a motor vehicle accident. His injuries included: skull base fracture; diffuse axonal brain injuries, bilateral lung contusions with left side diaphragmatic rupture and dissection of descending aorta. 

On admission to ICU he was sedated and mechanically ventilated. His blood pressure was 134/54 mmHg and pulse was 69 beats/min. Due to a potentially high risk of rupture and bleeding from the aortic dissection, the patient underwent urgent endovascular stenting. The procedure was completed successfully uneventfully. 

For the following two weeks the patient was treated for severe traumatic brain injury. Despite adequate sedation and analgesia the patient had become extremely hypertensive (blood pressure 215/100 mmHg). CT imaging and ICP monitoring did not reveal any signs of elevated intracranial pressure, or signs of herniation.

**Figure 1:**
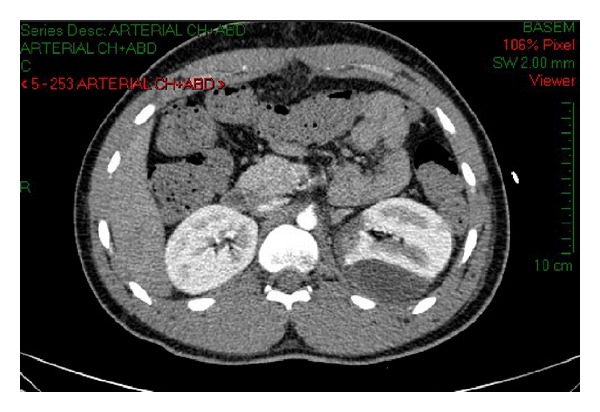
Ct abdomen showed subcapsular renal hematoma around left kidney (see black arrows).

A detailed clinical workup was performed, and included a repeated abdominal CT, which demonstrated a remarkable subcapsular hematoma of the left kidney (Figure 1), with no active bleeding. There was no biochemical evidence of kidney dysfunction or reduction in GFR.

The patient was started with and angiotensin converting enzyme inhibitor and an alpha-beta blocker *Labetalol. *Due to persistent hypertension, despite optimal medical treatment a surgical approach (capsulotomy or nephrectomy) was considered. Due to refusal of the patient's proxy, surgery was not performed. Thus, ultrasound guided hematoma drainage was done with a total of 80 mL of sanguinous fluid withdrawn. Gram stain analysis did not detect any microorganism. Antihypertensive therapy was continued after the procedure. Further workup for primary hypertension which included a cardiac echocardiogram and vascular ultrasound did not reveal abnormalities.

After one month of ICU hospitalization the patient was discharged for rehabilitation for rehabilitation. The patient remained dependent on anti-hypertensive medications for 6 months. Due to a poor response to ACE inhibitors, the mainstay of treatment was comprised of both beta-blocking agents and calcium channel blockers. Plasma renin concentrations were not measured.

## 3. Discussion

The main therapeutic strategy of Page Kidney is based on controlling the associated hypertensive disorder [1, 2]. In addition to trauma, Page Kidney has been described patients with renal tumor, renal cyst rupture, vasculitis, anticoagulation (warfarin) and various interventional procedures (kidney biopsy, lumbar anesthetic block etc) [6–9]. The majority of trauma cases are characterized by lower systolic blood pressure (Smyth et al. [2]) when compared to non-trauma related causes. In our patient, the hypertensive disorder was characterized with extremely high systolic blood pressure (systolic blood pressure over 200) resistant to single drug therapy. This is in contrast to previously published clinical data demonstrating that most trauma cases are successfully managed by ACE inhibitor therapy only. Recent findings show that surgical intervention may be avoided by an adequate medical therapeutic approach. Persistent hypertension has been treated by several alternative approaches-percutaneous and open hematoma drainage, capsulotomy and total nephrectomy. Dopson et al. [1] demonstrated good clinical response after surgical intervention (capsulotomy and nephrectomy) especially in motor vehicle accident and sport trauma cases unresponsive well to primary antihypertensive therapy. Importantly, in their report, most trauma cases became normotensive postoperatively. In the present case, the patient underwent ultrasound-guided drainage without further surgical intervention and remained hypertensive chronically. Mathew et al. [10] suggested that ultrasound percutaneous drainage might be insufficient for small sub capsular hematomas or may not be capable of removing well-organized hematomas. Finally, our patient was required multidrug therapy to control his high systolic blood.

In conclusion, we suggest that trauma induced Page Kidney may result in extremely elevated blood pressure unresponsive to single drug therapy. In such cases, an early surgical approach may prove to be beneficial, in light of the presence of high and uncontrolled systemic blood pressure parameters. This may prevent persistent hypertension later.
